# Resolution of the three dimensional structure of components of the glomerular filtration barrier

**DOI:** 10.1186/1471-2369-15-24

**Published:** 2014-02-01

**Authors:** Kenton P Arkill, Klaus Qvortrup, Tobias Starborg, Judith M Mantell, Carlo Knupp, C Charles Michel, Steve J Harper, Andy HJ Salmon, John M Squire, Dave O Bates, Chris R Neal

**Affiliations:** 1 Nanoscale Physics Research Laboratories, School of Physics and Astronomy, University of Birmingham, Birmingham, UK; 2 CFIM, Pannum Institute, Copenhagen, Denmark; 3 Wellcome Trust Centre for Cell Matrix Research, Faculty of Life Sciences, University of Manchester, Manchester, UK; 4 Wolfson Bioimaging Facility and School of Biochemistry, University of Bristol, Bristol, BS8 1TD, UK; 5 Optometry and Vision Sciences, Cardiff University, Cardiff, UK; 6 Bioengineering, Imperial College, London, UK; 7 School of Physiology and Pharmacology, University of Bristol, Bristol, UK; 8 Cancer Biology, Division of Oncology, School of Medicine, University of Nottingham, Queen’s Medical Centre, Nottingham NG2 7UH, UK

**Keywords:** Urinary space, Podocyte, Glycocalyx, GFB, Tomography, Serial block face (SBF), Focused ion beam (FIB), Electron microscopy, Glomerulus, 3D reconstruction

## Abstract

**Background:**

The human glomerulus is the primary filtration unit of the kidney, and contains the Glomerular Filtration Barrier (GFB). The GFB had been thought to comprise 3 layers – the endothelium, the basement membrane and the podocyte foot processes. However, recent studies have suggested that at least two additional layers contribute to the function of the GFB, the endothelial glycocalyx on the vascular side, and the sub-podocyte space on the urinary side. To investigate the structure of these additional layers is difficult as it requires three-dimensional reconstruction of delicate sub-microscopic (<1 μm) cellular and extracellular elements.

**Methods:**

Here we have combined three different advanced electron microscopic techniques that cover multiple orders of magnitude of volume sampled, with a novel staining methodology (Lanthanum Dysprosium Glycosaminoglycan adhesion, or LaDy GAGa), to determine the structural basis of these two additional layers. Serial Block Face Scanning Electron Microscopy (SBF-SEM) was used to generate a 3-D image stack with a volume of a 5.3 x 10^5^ μm^3^ volume of a whole kidney glomerulus (13% of glomerular volume). Secondly, Focused Ion Beam milling Scanning Electron Microscopy (FIB-SEM) was used to image a filtration region (48 μm^3^ volume). Lastly Transmission Electron Tomography (Tom-TEM) was performed on a 0.3 μm^3^ volume to identify the fine structure of the glycocalyx.

**Results:**

Tom-TEM clearly showed 20 nm fibre spacing in the glycocalyx, within a limited field of view. FIB-SEM demonstrated, in a far greater field of view, how the glycocalyx structure related to fenestrations and the filtration slits, though without the resolution of TomTEM. SBF-SEM was able to determine the extent of the sub-podocyte space and glycocalyx coverage, without additional heavy metal staining. Neither SBF- nor FIB-SEM suffered the anisotropic shrinkage under the electron beam that is seen with Tom-TEM.

**Conclusions:**

These images demonstrate that the three dimensional structure of the GFB can be imaged, and investigated from the whole glomerulus to the fine structure of the glycocalyx using three dimensional electron microscopy techniques. This should allow the identification of structural features regulating physiology, and their disruption in pathological states, aiding the understanding of kidney disease.

## Background

**Figure 1:**
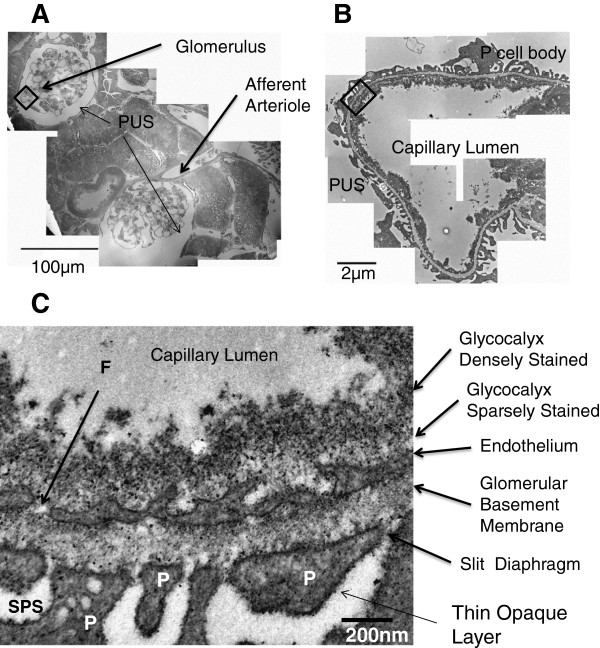
**Structure of the glomerular filtration barrier.** Transmission electron microscopy of a perfusion fixed rat glomerulus with addition of LaDy GAGa stain (section 80 nm thick). **A**: A montage of low magnification images depicting two glomeruli. **B**: A montage of higher magnification images depicting a glomerular filtration capillary (boxed in **A). C**: A high resolution (24,500 times magnification, boxed in **B**) of the five layers of the glomerular filtration barrier. There are two layers of staining (dense and sparse) present in this example of endothelial glycocalyx. A thin layer of contrast outside of the podocyte membrane is also commonly present. P = podocyte, C = capillary lumen, PUS = periphery urinary space, F = fenestration, SPS = sub-podocyte space.

Filtration of fluid and solutes in the kidney occurs through the renal glomerulus, a structure in the renal cortex consisting of a knot of microvessels sitting between afferent and efferent arterioles (Figure 1). The glomerular filtration barrier (GFB) retains most of the plasma proteins (e.g. albumin) in the vasculature, allowing the filtration of all small solutes, but maintaining the colloid osmotic pressure of the plasma, enabling fluid retention in the rest of the body. The GFB has classically been described as consisting of three layers –the podocyte foot processes, the basement membrane and the fenestrated endothelium [1]. In recent years however, two additional layers to the GFB have been realised [2]. On the vascular side, a fine meshwork of glycosaminoglycans covers the endothelial layer, including the fenestrations [3-6]. This glycocalyx contributes to the permeability properties of the GFB [7-9]. On the urinary side of the GFB the fluid can pass across the filtration slits into an area under a podocyte cell body called the subpodocyte space (SPS). This area is bounded by the podocyte cell body on one side and the podocyte foot processes on the other. Along with the SPS there are two further types of urinary space: the interpodocyte space (IPS, between podocyte cell bodies) and the peripheral urinary space (PUS) between the glomerular edge and the walls of the glomerular capsule [10,11]. Narrow exit pores between the SPS and IPS and the PUS restrict flow away from the GFB.

The current view on the shape and position of the urinary spaces inside the renal glomerulus assumes that the major resistance to flow of fluid occurs at the classical three layered GFB [6]. However, there is mounting evidence that podocyte cell bodies and processes which cover a substantial portion of the GFB make a restrictive SPS. The SPS appears restrictive to fluid flow and shows a change in size depending on the pressure applied to the glomerular capillaries [10,12]. Also, SPS shows an ability to retain macromolecules and alter fluid flowing through it [11]. The SPS was first identified in 1950 [13], re-described in 1965 [14] and analysed in detail more recently [10,15]. Mathematical modelling has shown that the SPS can act as a resistance to fluid flow [12], and more recently that the increase in fluid exchange induced by vascular endothelial growth factor over-expression across the glomerulus was at least in part explained by the reduction in the SPS coverage of the GFB revealing a greater free surface area of the GFB for unrestrictive fluid filtration [16].

A detailed analysis of the SPS from 6 glomeruli of 4 rats showed that 50-60% of the GFB was covered by SPS. The IPS conduits inside the glomerulus feed filtrate away from the ‘free’ GFB or from an SPS exit region and the peripheral area that collects filtrate from the glomerular edge forms the PUS (though this has very narrow dimensions when the vascular oncotic and hydrostatic pressures used at fixation have physiological values [12]). However, these definitions are only as good as the serial sectioning and reconstruction techniques will allow. Greater runs of serial sections allow a better appreciation of glomerular structural relationships. So far with conventional transmission electron microscopy (TEM) sectioning and reconstruction only one complete SPS has been reconstructed from 120 sections through parts of a rat glomerulus [10]. All other data have been reported on lower numbers of sections of incomplete SPS. Longer sectioning runs for better appreciation of the ultrastructure are required, but are very time consuming, labour intensive, technically difficult, and expensive.

Many of the same issues apply to investigating the endothelial glycocalyx but at much higher magnification. Single EM sections of the glycocalyx do not provide details of the overall glycocalyx coverage of the endothelial cells. They cannot show how the glycocalyx sits over the fenestrations or the relationship with the underlying foot processes, or whether the glycocalyx coverage is similar in different areas of the glomerulus. Furthermore the detailed architecture of the glycocalyx requires three-dimensional imaging of the fibre matrix network that provides resistance to flow in both the horizontal (*en face* to the endothelial surface) and vertical (from the lumen through to the endothelial surface or pores) direction.

Serial block face scanning electron microscopy (SBF-SEM) images a resin embedded tissue block surface and uses an inbuilt diamond knife to remove a thin layer of resin embedded tissue (70-150 nm) prior to re-imaging. By using backscattered electron intensity contrast, little topographical information is revealed but atomic number contrast arises. Thus, an exposed surface or area of a resin block can be imaged. This image when inverted gives a micrograph similar to a transmission electron micrograph. This is in essence the same as taking many TEM micrographs from serial sections, and though it is relatively low resolution (in TEM terms) can be undertaken automatically and without operator interference.

Focused ion beam milling scanning electron microscopy (FIB-SEM) is a similar technique but after backscatter imaging the block face is milled with an ion gun, vaporising a precise layer (10 nm) of tissue and resin from the block, prior to reimaging the surface. This method can determine many ultra-structural properties and importantly the depth of field (z-direction) is comparable to the xy dimension. Although, in principle, it does not have a resolution advantage over the SBF-SEM in the xy plane, the depth removed is finer and with no diamond knife mechanical interference there are fewer surface charging artefacts after Focused Ion Beam milling.

Finally, transmission electron tomography (Tom-TEM) can be used to reconstruct an image of a ~300 nm thick tissue section from images of the section at multiple angles of rotation in a transmission electron microscope. Images are combined to form a three-dimensional reconstruction of small regions of the section with nm resolution in the x, y and z directions.

Here we evaluate these three complementary techniques of 3D electron microscopy in kidneys derived from the same animal to investigate their potential for resolving the three-dimensional organisation and structures of the glomerulus. Additionally, a human kidney has been used to demonstrate SBF-SEM.

## Methods

### Tissue fixation and processing

All rat experiments were conducted in accordance with UK Home Office regulations.

Rat Kidney: Intracardiac perfusion of a glutaraldehyde fixative containing lanthanum and dysprosium to stain the glycosaminoglycans (Lanthanum Dysprosium GlycosAminoGlycan adhesion method (LaDy GAGa)) was carried out as previously described [17]. A male Sprague Dawley rat was injected with a lethal dose of 0.7 ml/kg sodium pentobarbitone (Lethobarb, Ayrton Saunders Ltd UK), the thorax promptly opened and the left side of the heart perfused at 100 mmHg pressure with a flush solution of HEPES buffered mammalian Ringer containing 0.5% LaNO_3_.6H_2_O, 0.5% DyCl_3_.6H_2_O, (room temperature, pH 7.3). Heparin anticoagulant was not used because of potential cross-reaction with the lanthanides in solution. Flushing with a 3 ml bolus was followed by 100 ml of 2.5% glutaraldehyde, 2% sucrose in the same solution as above (room temperature, pH 7.3). Tissues started to stiffen within 30 seconds. Samples 1 mm in diameter were cut from each kidney and stored at 4ºC for 2 days to several weeks in buffered glutaraldehyde. Tissues were washed with HEPES buffer and transferred to 0.1 M sodium cacodylate buffer (pH 7.3), postfixed in 1% OsO_4_ in 0.1 M cacodylate buffer (pH 7.3) for 1 h and rinsed in 0.1 M sodium cacodylate buffer (pH 7.3). After Osmication, tissues were washed in distilled water, Fixed/stained with 2-3% aqueous uranyl acetate [~12 hrs, 4ºC], dehydrated in a graded series of ethanol, infiltrated with Araldite resin mixtures with propylene oxide and Araldite resin alone and then embedded in Araldite resin (Agar Scientific, UK).

A Human tissue block was prepared from an unused transplant kidney by perfusion fixation. However, additional aqueous heavy metal steps were applied using an adapted West *et al*[18] protocol (See Additional file 1). Dehydration and resin infiltration used the rat protocol. Research involving human subjects was performed with appropriate consent, and after review by UK research ethics committee (study approval 07/H0102/45) in accordance with UK legislation and the Helsinki Declaration.

### Tissue block preparation

#### ***SBF-SEM***

Tissues blocks with glomeruli of interest were demarcated and each trimmed to approximately a 500 μm × 500 μm block face with less than 2 mm depth using a razor blade. The blocks were mounted using cyanoacrylate adhesive (Permabond) onto a 3 mm head diameter aluminium mounting pin. The pin head and block were sputter-coated with gold ready for SEM.

**Figure 2:**
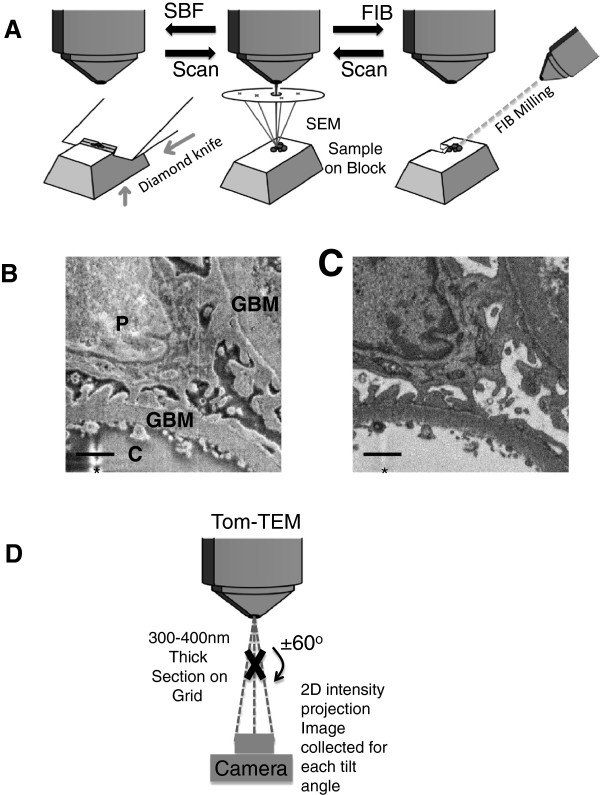
**Illustration of methods for SBF-SEM, FIB-SEM and Tom-TEM. A**: Illustration of the imaging procedure for SBF-SEM and FIB-SEM. The scanning electron beam (centre) raster scans a specific area of the surface and backscattered (or secondary electron) micrographs are collected. For SFB-SEM (left) the sample is raised and then cut with a diamond knife, for FIB-SEM (right) a focused ion beam mills a layer from the surface before returning to the scanning mode. **B:** Secondary Electron and **C:** Backscattered Electron Micrographs from SFB-SEM. P = Podocyte, GBM = Glomerular basement membrane and C = Capillary Lumen. * Knife mark is less visible in **C** than **B**. Scale Bars = 1 μm **D**: Electron tomography (Tom-TEM) The electron beam passes through the sample. The sample is tilted so that images are taken at different angles without moving the detector (camera). The images at known viewing angles can then be reconstructed, for example using weighted back-projection, to produce a 3D image of the sample.

The trimmed mounted block was attached to the chuck and the resin embedded glomeruli were sectioned using a Gatan 3view ultramicrotome within an FEI Quanta 250 scanning electron microscope (SBF-SEM). This technique, based on the work of Denk and Horstman [19] allows a backscattered electron image of the block face to be collected after each diamond knife cut (Figure 2A).

Once set up inside the microscope an automatic routine imaged the surface, advanced the specimen and sectioned the block face removing a 150 nm thick resin section (50 to 200 nm thickness is possible for resin embedded material). The knife was moved away from the block and the surface re-imaged. The sequence of advance, cut, clear (the block), image was repeated until all the images were acquired. Multiple images were stacked together to build up a three-dimensional reconstruction of the original sample. The data stack was imaged using backscattered electron detection at 2.5 kV, on a 2048 × 2048 array with 25 μs dwell time at a chamber pressure of 0.56 mbar in a water vapour atmosphere to earth and reduce sample charging. Each cut was a 150 nm slice. The detected depth is expected to be under 10 nm where there is stain. Five hundred images were taken giving an overall volume of 84 μm × 84 μm × 75 μm. This relates to a 41 nm × 41 nm × 150 nm voxel size.

#### ***FIB-SEM***

Firstly, an 80 nm thick survey section was imaged with a Philips 100CM TEM to map the surface of the block. The block was then trimmed with an ultramicrotome to place the region of interest (ROI) at the edge of the block. The ROI with surrounding resin was removed from the resin block, stuck to a metal pin with conductive carbon cement and then sputter-coated with gold to increase conductivity.

Once the ROI was identified by SEM, the block was tilted to 52º and trenches milled with a focused gallium ion beam (Ga^+^) either side of the image area. A 1 μm layer of platinum was applied along the expected ROI cutting area by gas injection negating the requirement for a water vapour atmosphere to reduce charging.

The area was imaged at 52° and then 10 nm resin depth was milled away by the Ga^+^ ion beam. The image–mill sequence was repeated for the required depth (Figure 2A). Backscattered electron contrast imaging was used at 4096 × 3536 pixels for 10 μs dwell time, resulting in voxel dimension of 1.04 nm × 1.32 nm × 10 nm. The pressure and accelerating voltage were 2.54 × 10^-10^ mbar and 2 kV respectively.

#### ***Tom-TEM***

Tom-TEM was carried out as previously described [17]. In brief, 80 nm thick survey sections used to locate vessels of interest and serial 300 nm thick sections for tomography were cut with a Reichert-Jung Ultracut E microtome and mounted on Pioloform support films on slot grids. Gold beads of 10 nm diameter (fiducial markers: Aurion, Wageningen, Netherlands) were carefully added to both sides without dilution. The gold bead suspension was sonicated to reduce coagulation and grids were placed in a drop of suspension for 20 minutes, turning the grid over after 10 minutes, and then excess liquid was removed with blotting paper. Tom-TEM was performed with an FEI T20 200 kV electron microscope with a Fishione 2040 dual tilt holder. Once in the microscope the section was pre-shrunk using methods described in Mantell *et al*[20] adapted from [21]. Briefly, the section was aligned under low dose protocols, tilted to 45° and 60 one second exposure micrographs were taken at the intended beam intensity. By observation of movement of the fiducial markers the original thickness could be calculated. GFB tomogram micrographs were imaged with the section tilted in increments of 1º in a minimum range between -60° and +60° using an Eagle 4096 × 4096 CCD camera (FEI) at 29,000× magnification (0.37 nm/pixel). The exposure time was 2.0 s for the 0° view and was increased for each degree up to 3.2 s for ±60° views to account for the increase of section thickness at higher viewing angles (Figure 2D). FEI automated tomography acquisition software (with a small degree of manual intervention) was used to acquire the images into a 16-bit MRC file stack. The process was repeated for the same section location with a second tilt axis at 90° (in the x-y plane) to the first. The two tilted image series were reconstructed into a single 3D density image stack using IMOD tomogram reconstruction software [22,23]. It was noted that a careful auto-focus set-up and a steady beam intensity, though not imperative, significantly improved the ease of reconstruction.

### Analysis

Image analysis was performed with ImageJ (NIH) [24] but additionally a combination of IMOD [23], Amira (Visage Imaging) and Fiji [25] were also used. Tom-TEM reconstructions and general large file data stack manipulations of FIB-SEM and SBF-SEM were performed with IMOD. Amira was used for segmentation. Fiji was used for image registration, 3D visualisation and other analysis.

To determine glycocalyx fibre spacings in the reconstructed tomogram an autocorrelation method was used from previous publications [26,27]. In brief: X image areas (150 nm by 150 nm) containing glycocalyx were chosen in a reconstruction slice. These were extracted at the same coordinates for every slice if the area contained glycocalyx. To these areas a taper to the intensity mean was added followed by a fast Fourier transform. After this the autocorrelation function was performed leaving a correlation map in real space. Radial profiles were then taken in a direction parallel to the membrane. All positive peaks in the profile were found using the second differential where the distance is a predominant spacing. The peaks were only used if they were above a threshold value determined by the second differential value of control (blank resin) areas.

## Results

### Ultrastructure of the glomerular filtration barrier

The complex nature of the GFB is demonstrated by classical transmission electron microscopy (TEM) in Figure 1A where a low power image of two rat glomeruli are seen in cross section after LaDy GAGa staining. The higher power view of a single capillary (Figure 1B) shows the presence of the podocyte, foot processes, endothelium, fenestrations and peripheral urinary space. At 24,500 times microscope magnification (Figure 1C) the detailed structure of the GFB reveals that a complex glycocalyx overlies the endothelium, with a high contrast layer sitting above a less electron dense layer just above the fenestrated endothelium. The glycocalyx layer revealed by this staining is 200-400 nm thick. These images also reveal a thin layer of contrast lining the podocyte foot process cell membrane, indicative of a glycocalyx on the urinary side, although this is an order of magnitude thinner as revealed by this perfusion fixation and staining technique.

### SBF-SEM

The benefit of using an SEM is that several electron imaging modes can be acquired at the same time (Figure 2B/C). Figure 2B shows secondary electron and Figure 2C the backscattered electron intensity image from the same block face. Secondary electrons are low energy electrons detected from the surface influenced by topographical information along with stain density. In the image a small knife mark (asterisked) is visible 1/4 of the way across the image running top to bottom. Some beam damage in the empty resin in the bottom left hand corner is also visible indicating that the beam intensity limit of this patch of resin has been reached. The imaging conditions were therefore carefully chosen to balance image quality with speed of acquisition and volume of beam interaction, defining the resolution limit. The back-scattering electron signal (Figure 2C) is predominantly affected by the atomic number of the atoms in the sample, resulting in greater visualisation of the stained material within the sample. By inverting the signal intensity of the backscattered electron micrographs an image similar to a standard TEM micrograph of a stained section can be formed. This method contrasts with Tom-TEM, whereby the sample is tilted on a stage and the images reconstituted to provide a three dimensional view through the section (Figure 2D).

**Figure 3:**
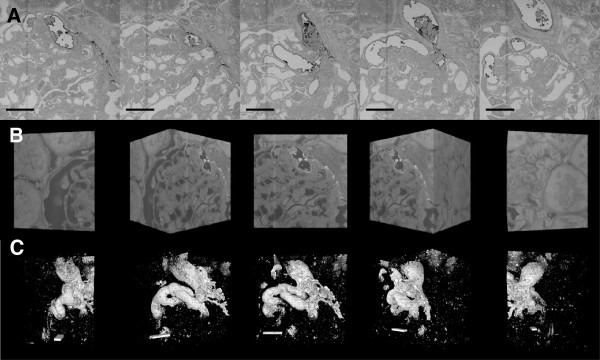
**SBF-SEM contrast imaging of glomerular glycocalyx. A**: Section sequence (every 7.5 μm) from raw data stack (71 μm × 71 μm × 84 μm) The LaDy GAGa stained components can be seen as dark regions adjacent to the lower contrast of a conventionally processed TEM resin block (Additional file 2: Movie S3A). **B**: An inverted contrast image sequence (every 40^o^) of a 3D surface projection of the same Image Stack as above (Additional file 2: Movie S3B). **C**: The 3D surface projection has been intensity thresholded to reveal the LaDy GAGa (glycocalyx) stained vessels and structures (Additional file 4: Movie S3C). Scale Bars = 20 μm in length.

Figure 3A shows six images from a stack of 500 serial sections (150 nm section thickness) through a rat glomerulus stained by LaDy GAGa (Additional file 2: Movie S3A). These representative images indicate that SBF-SEM could be used to image the depth of a whole glomerulus. In this glomerulus, the lanthanum and dysprosium was perfused into the first afferent vessels in the glomerulus and was chosen as it demonstrated clearly the 3D nature of the vessels. The dark staining of the glycocalyx can be seen in the afferent arteriole and the first few glomerular vessels. Figure 3B shows a rotated 3D-reconstruction of the images (see Additional file 3: Movie S3B). With the glycocalyx highlighted and extracted from the image (Figure 3C and Additional file 4: Movie S3C) the glycocalyx coverage of the initial vessels is seen to be extensive and easily defined in three dimensions.

**Figure 4:**
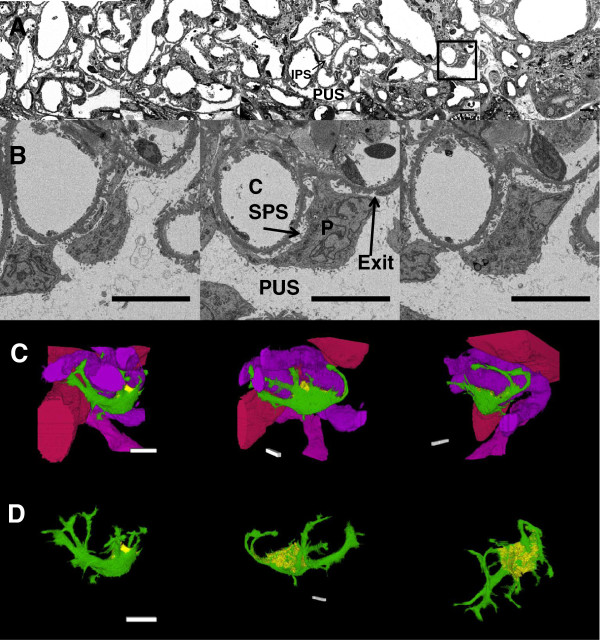
**Serial block face scanning electron microscopy of a human glomerulus. A:** Micrographs of complete field of view, slices 15 μm apart (z) at the edge of a glomerulus (Additional file 5: Movie S4A). **B** Micrographs of a podocyte (P) from inset in **A** to illustrate the sub-podocyte space (SPS). C = Capillary lumen, PUS = periphery urinary space, IPS = inter-podocyte space, and Exit is an exit region for an SPS (Additional file 6: Movie S4B). **C:** The same podocyte as in **B** and adjacent capillaries after highlighting and reconstructing (Additional file 7: Movie S4C). **D:** as **C:** The podocyte is differently oriented, and capillaries have been removed to display the SPS (Additional file 8: Movie S4D). Purple/Pink = Capillaries. Green = Podocyte cell body. Yellow = SPS (under the main cell body). Scale bar is 10 μm in all cases.

To determine whether this technique could be used to reconstruct human glomeruli, a perfused human kidney was subjected to SBF-SEM. The resulting images clearly show SPS, IPS and PUS in the kidney (Figure 4). Figure 4A shows image slices from the raw data (Additional file 5: Movie S4A and Additional file 6: Movie S4B). Figure 4B shows image slices through a podocyte cell body. Figure 4C and 4D show the reconstruction of the podocyte cell body with and without capillaries. The SPS has been coloured yellow to aid visualisation (Additional file 7: Movie S4C and Additional file 8: Movie S4D). Nine SPS areas from under six podocytes were tracked for their entirety (for details see Table 1). Any exit to another urinary space was noted, the height of SPS exit regions ranged from less than 41 nm (pixel size) to 2000 nm. Most were approximately the same as the height of the SPS, rather than a specific constriction. Some did have no obvious exits, though areas where an exit less than 40 nm could be likely were observed.

**Table 1:** Measured Sub-Podocyte Space (SPS)

**SPS**	**Exit region width /nm**	**Approximate SPS Length/um**	**Depth/nm**
1	40	12	600
2	200	9	200
2a	<150	9	850
3	1200	6	400
4	2000	6	600
5	1300	2	500
6	250	2	300
7	300	9	1800
8	40	3	80
9	0	6	500

The largest of the exits to Inter-Podocyte Space or Periphery Urinary space, the approximate length under the podocyte and depth (between capillary and podocyte body) of the SPS are also shown. Note: there were two measurable SPSs under one cell 2/2a.

**Figure 5:**
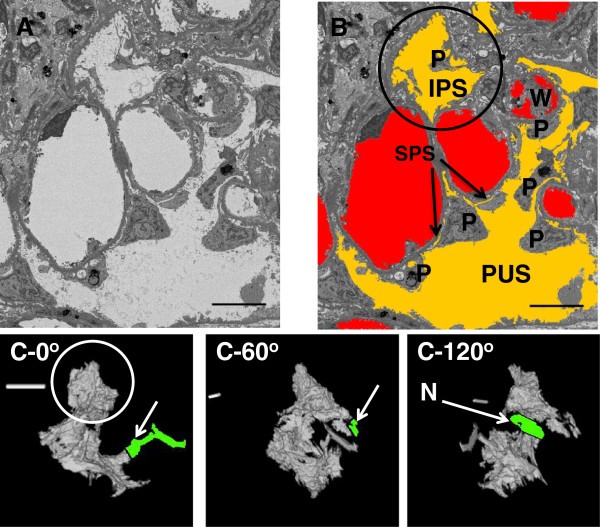
**The IPS in human glomeruli. A**: Example SBF-SEM micrographs of the human glomerulus (Additional file 9: Movie S5A). **B**: The same micrograph as **A** but with capillaries (red) and urinary spaces (orange) highlighted and example podocytes (P) and white blood cell (W) labelled. The inter-podocyte space (IPS) and periphery urinary spaces (PUS) are shown (Additional file 10: Movie S5B). **C**: The extracted IPS as a volume at different angles. The circle is the IPS marked by the circle in **B**. The arrows and highlights in **C-0** and **C-60** point to the two connections with larger IPS. The arrow in **C-0** is a substantial connection (circa 5 μm wide, though this channel is filled with podocyte cell bodies), but the connection between this IPS and the circled IPS is a narrow (<1.5 μm) neck (label: N in **C-120**) and is tortuous with podocyte parts occluding the channel. The other connection to the circled IPS (arrow in **C-60**) is highly tortuous and very narrow channel if it connects at all. See Additional file (Additional file 11: Movie S5C) for the whole image and reconstruction. Scale Bars = 10 μm.

Figure 5 shows a reconstruction of a human IPS (approximate volume 70 μm^3^). Figure 5A (Additional file 9: Movie S5A) is an area of an image from the sequence and this has had the relevant areas coloured in Figure 5B (Additional file 10: Movie S5B). Reconstruction indicates that this space is complex, interweaving and tortuous, with narrow exits to the peripheral urinary space. This space has been seen in section before but to fully define these areas serial section stacks would have been prohibitively long (TEM) or too low in resolution (light microscopy). The IPS studied within the image stack (illustrated in Figure 5C) showed evidence of passages to larger areas of PUS of only 500-1000 nm wide represented as a tortuous ribbon (Additional file 11: Movie S5C). These routes interweaved with podocytes cell bodies and foot processes on capillary walls. Furthermore there was a substantial bulb-like space, which appears to have a connection of less than 100 nm at one side and only one narrow ribbon-like connection from the other side (‘N’ in Figure 5C).

### FIB-SEM

**Figure 6:**
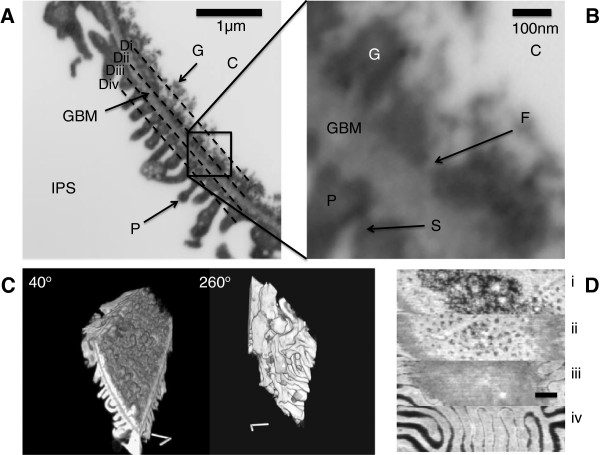
**FIB-SEM micrographs of a glomerular capillary wall. A** and **B:** The glomerular filtration barrier using backscattered electron imaging (Additional file 12: Movie S6A). Labels: (C) capillary lumen, (IPS) interpodocyte space, (P) podocyte foot process, (GBM) glomerular basement membrane, (G) glycocalyx stained with LaDy GAGa, (F) fenestration and (S) slit diaphragm. **C:** A 3D surface image of the xyz stack (Scale Bar is 1 μm) (Additional file 13: Movie S6B). **D:** A set of stills through the glomerular filtration barrier (Approximately perpendicular to and indicated by dashed lines in **6A**). i) Glycocalyx, ii) fenestrations, iii) glomerular basement membrane, iv) podocyte foot processes (Scale Bar is 500 nm).

Figure 6 demonstrates reconstruction of the glomerular filtration barrier using FIB-SEM. Figure 6A (Additional file 12: Movie S6A) shows a single image at low power of a filtration region with no overlying SPS. A magnified region of Figure 6A is shown in Figure 6B. The LaDy GAGa staining was clear and the depths of glycocalyx could be estimated. The three dimensional reconstruction using this technique on 295 sections demonstrates both the variability in the glycocalyx surface (Figure 6C) which appears knobbly and irregular and the complexity of the podocyte foot processes on the other side showing complex overlapping structures (see Additional file 13: Move S6B). Sections taken through the GFB from the lumen (Figure 6D) show the potential for FIB-SEM in determining the spatial relationship between GFB components revealing the relative dispositions of glycocalyx, fenestrations, GBM and foot processes.

### Tom-TEM

Using Tom-TEM a volume of 2 μm × 2 μm × 300 nm of the GFB of a rat glomerulus perfused with the LaDy GAGa technique was reconstructed. The resolution was 1.8 nm in the xy directions, meaning that cell membranes could be easily distinguished. The reconstruction resulted in a depth (z) resolution of 4.5 nm due to the missing acquisition wedge and 25% section shrinkage.

**Figure 7:**
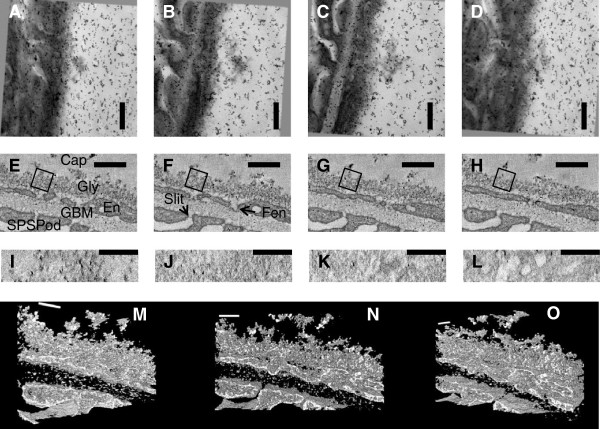
**Tomographic reconstruction of rat glomerular capillary glycocalyx using LaDy GAGa staining on a 300 nm thick section. A-D:** raw transmission electron microscopy at -60^o^,-30^o^,30^o^ and 60^o^ respectively (Additional file 14: Movie S7A). **E-H:** reconstructed images of glycocalyx in cross-section 30 nm apart (Additional file 15: Movie S7B). There is the appearance of a gap between the glycocalyx (Gly) and the endothelial cell (En). Small holes can be seen throughout Gly, these are gaps between the fibres. Boxes are examples of areas used for spacing analysis. **I-L:** are the reconstruction viewed perpendicular to the membrane. **I:** is at the top of the glycocalyx, **J:** is 70 nm from the top, **K:** is 140 nm from the top and **L:** is within the endothelial layer (fenestrations can clearly be seen). **M-O:** Are stills from the 3D projection (Additional file 16: Movie S7C). **A-L:** scale bar is 300 nm. **M-O:** scale bar is 150 nm. Labels: Cap = capillary lumen, Gly = glycocalyx, Fen = Fenestration, GBM = glomerular basement membrane, SPS = subpodocyte space, Slit = Slit Diaphram (the diaphragm is discernable), Pod = Podocyte (foot process), En = endothelial cell.

Figure 7 demonstrates the raw image data and the reconstruction. As the section was rotated in the EM goniometer, differing views of it were imaged (Figure 7A-D, Additional file 14: Movie S7A). Reconstruction at a single slice level across the GFB (Figure 7E-H, Additional file 15: Movie S7B) demonstrates clearly the SPS foot processes, basement membrane, endothelial cell with fenestrations, and glycocalyx. The glycocalyx coverage of the endothelium is complete being 100-200 nm in depth, and the stained fibres could be resolved. There is a 25 nm thick layer of reduced staining between the membrane and denser glycocalyx layer. Auto-correlation to determine the fibre spacing [17,26,27] of the densely stained glycocalyx from 947 image areas over 225 slices (each area approximately 150 nm by 150 nm as in Figure 7E-H) showed an expected mean fibre spacing of 20 nm but no lattice could be determined. Reconstruction in the en-face view of the glycocalyx was also possible using this technique (Figure 7I-L) and the variation in local coverage can be observed. The three dimensional reconstruction of the whole glycocalyx layer and GFB is shown in three different views in Figure 7M-O (Additional file 16: Movie S7C).

## Discussion

The fine three-dimensional structure of the GFB is critically related to its function, but the vast majority of studies on the function of the GFB have used imaging techniques that do not reconstruct it. The structure of the glycocalyx provides a meshwork that acts as a resistance to flow which depends on the three dimensional arrangements of the fibres, and their position relative to underlying highly porous area such as endothelial clefts and fenestrations in the endothelial cells. The basement membrane filtration capacity depends on the 3D arrangement of its constituent fibres, but the alignment of the flanking podocyte slit diaphragms and endothelial fenestrations is important but completely unknown. The three dimensional network of the sub-podocyte space and linking interpodocyte space has not yet been fully defined. However the dimensions of these urodynamic pathways should inform on their physiological function. It is therefore clear that techniques for the assessment of glomerular structure are required and we have shown that multiscale 3D glomerular reconstruction is possible and relatively efficient using SBF-SEM, FIB-SEM and Tom-TEM.

The staining employed gave a glycocalyx height of 100-400 nm, depending on imaging modality. This is more comparable with light microscopy than previous EM values (*circa* 50 nm e.g. [5]). Though we believe LaDy GAGa staining is an improvement over techniques using tannic acid (a mordent), primarily because these lanthanide ions are small enough to enter the glycocalyx and stain fibres, we cannot be sure that we observe the actual fibre, or just the surrounds of it. Although fast freezing techniques with freeze substitution can improve structural preservation compared to aldehyde fixation (e.g. Hawes *et al*[28]) they are of less use when in-vivo capillary pressure is required or specific filtration conditions need to be preserved during fixation. The glycocalyx fix/stain is delivered to the capillary wall as rapidly as possible with perfusion fixation and fixes under conditions of flow, pressure and filtration.

### SBF-SEM

Once set up the total run time to section and image 500 slices was 14.5 h on the SBF-SEM, without human intervention. Some of the serial images were out of focus and this was assumed to be due to a small sample fleck interfering with the beam. Even though an extensive staining procedure was required to induce enough electron density within the sample, the overall image quality appeared very good and no differences in ultrastructure could be attributable to the SBF-SEM technique. To create and document this amount of data by traditional serial ultrathin sectioning methods is possible though highly expensive in technical man-hours and EM time. The lanthanum and dysprosium stained glycocalyx had good contrast in the rat glomerulus, but the tissue with no extra heavy metal staining did not, therefore extra heavy metal doping was required to observe general tissue. Even without an improved backscatter detector, SBF-SEM achieved good sub-cellular resolution with a >70 μm cube of tissue. Figure 4 also emphasizes the possibilities of SBF-SEM in allowing identification of multiple features, rapid reconstruction and colour coding to reveal how structures and spaces relate to each other. There is no reason this reconstruction could not have been done for a depth of several hundred micrometres to encompass an entire human glomerulus, something totally impractical at this resolution with manual sectioning.

### FIB-SEM

The total run time on the FIB-SEM to section and image 300 slices was 9 h.

The FIB-SEM had better backscatter electron detection (primarily due to the detector quality) than the SBF-SEM used. This accounts for the improved contrast between the SBF-SEM and FIB-SEM on the perfusion fixed rat without the extra heavy metal staining procedure.

In FIB-SEM the estimated resolution was in the order of 25 nm (by fast Fourier transform) in both the x and y directions, though due to the tilt this was not the same number of pixels; oversampling has contributed to a 5 fold lower field of view than could be possible. The 10 nm serial removals of material in the z direction changed the micrograph noticeably indicating a 20 nm resolution, between images but beam damage and artefact is likely on thinner cuts. Unlike SBF-SEM where near perfect alignment of z images is intrinsic, the FIB-SEM required lateral alignment between successive images. However, no significant warping or rotation was observed.

The FIB-SEM differs from the SBF-SEM by having a thinner slice removal capability. This makes it more suited to resolving objects an order of magnitude smaller than in the SBF-SEM. There is also an improvement in signal to noise and resolution due to the improved charge dissipation of the smaller cut surface. The datasets shown were milled in from a glass knife-trimmed surface, but it is relatively straightforward to excavate anywhere from an edge cut into a trimmed block surface, though navigation may be cumbersome without a good surface map from a TEM.

The resulting reconstruction gives novel insights into the 3D nature of the glycocalyx over the filtration barrier. It has a knobbly forest-like appearance, and gaps to the membrane. Such gaps and macrostructure, if physiologically accurate would alter the way in which filtration is modelled and understood. More studies with different stains, or specific studies without staining (freezing techniques), would be needed to determine the level of artefact within this protocol and hence the physiological relevance.

### Tom-TEM

Almost all new TEMs can perform tomography, and mostly come with software designed to save user time. The dataset volume is not usually cubic, with the z thickness being approximately10-fold smaller than the xy directions (before shrinkage correction). The resolution is the highest of the three 3D modalities, approaching the limit of resin-embedded sections: the glycocalyx was clear and the fibre spacings discernible (Figure 6E-H), but the missing angles in the tilt series were a limiting factor in the z direction. A dual tilt tomogram (the section is rotated 90° about a central z axis and the tilt sequence repeated) results in reduced missing information. In this case the resolution was approximately half that of the x-y resolution before the corrections for shrinkage were made. For many applications the section shrinkage is not an issue but for quantitative measurements this needs to be corrected for. The main issue is that shrinkage is anisotropic across regions of tissue/resin/stained components in the x, y and z direction.

An issue with conventional electron microscopy and the elucidation of ultrastructure whether in normal or diseased tissues is that sampling error can be enormous. Small sections of small parts of tissues are observed and assumptions are made about ultrastructural relationships in the remaining unseen majority of the structure. Reconstruction of tissues has become easier through the advent of digital imaging and better ultramicrotomes, however, the process is still labour intensive. The techniques described here attempt to addresses this problem by greatly extending the effective volume of tissues analysed and correlating images at different resolutions.

## Conclusions

In conclusion this paper has demonstrated that SBF-SEM and FIB-SEM on perfusion fixed glomeruli have a practical niche between light microscopy and TEM, agreeing with other authors on other tissues [29]. They have at least 10 times better resolution than light microscopy sections, and due to the many interfaces and curved nature of glomeruli, 2-photon or confocal light microscopy would lose even more resolution. The SEM systems of 3D scanning therefore fit between TEM and light microscopy in resolution but with a far greater volume of tissue surveyed with the advantage of greatly reduced working man-hours. This is particularly suited to sub-cellular studies of convoluted capillary beds. TEM serial sections can have more than an order of magnitude better resolution, and, with tomography, three dimensional reconstructions are possible. However, a 500 nm section depth is deemed thick. The complementary electron microscopy techniques described here therefore allow a definitive reconstruction of the GFB, the glycocalyx, and subtypes of urinary space (SPS, IPS and PUS) within the glomerular capillary network as a whole entity. These data show the IPS and SPS not to be the free flowing urinary volume so often assumed. It does appear that the SPS exits and IPS exits are of similar dimensions. To investigate any physiological impact, however, a parameter study would need to be performed (i.e. varying pressure) and combined with other studies to determine any glycocalyx coverage on the urinary side.

## Competing interests

The authors declare that they have no competing interests.

## Authors’ contribution

All authors contributed to writing and discussion/interpretation of data, additionally: KPA: Tomography, idea to use FIBSEM and SBFSEM, all image analysis, significant writing. KQ operation and sample experimentation with FIBSEM. TS: Operation and sample experimentation of SBFSEM. JMM: Tomography and tomography shrinkage data/experimentation. CK: Tomography novel protocol advice. CCM: Contextual interpretation of results. SJH: Source of Human Kidney, and Kidney maintenance. AHJS: Rat surgery/fixation. JMS: Tomography novel protocol and image analysis advice. DOB: Contextual interpretation of results and significant writing. CRN: Rat surgery/fixation, almost all EM preparation and preparation protocols, block mapping and trimming, idea to use SBF on human samples and significant writing. All Authors read and approved the final manuscript.

## Pre-publication history

The pre-publication history for this paper can be accessed here:

http://www.biomedcentral.com/1471-2369/15/24/prepub

## Supplementary Material

Additional file 1 **Supplementary Material.** Additional heavy metal staining of human blocks. Click here for file

Additional file 2 **Video S3A.** SBF-SEM backscatter imaging of glomerular glycocalyx. 500 z slice (150nm) sequence from SBF-SEM backscatter imaging of glomerular glycocalyx. Scale bar= 20μm. Click here for file

Additional file 3 **Video S3B.** SBF-SEM backscatter imaging of glomerular glycocalyx (Inverted) contrast image rotation of 3D surface projection. Scale bar= 20μm. Click here for file

Additional file 4 **Video S3C.** SBF-SEM backscatter imaging of glomerular glycocalyx. The block segmented by intensity thresholding to reveal glycocalyx stain. Scale bar= 20μm. Click here for file

Additional file 5 **Video S4A.** Serial Block Face Scanning Electron Microscopy of a human glomerulus. 500 slice (150nm) sequence of complete field of view. Scale bar = 10μm. Click here for file

Additional file 6 **Video S4B.** Serial Block Face Scanning Electron Microscopy of a human glomerulus. As **Video S4A** but with colour assignment (segmentation). Purple/Pink = Capillaries. Green = Podocyte cell body. Yellow is SPS under the main cell body. Scale bar = 10μm. Click here for file

Additional file 7 **Video S4C.** Serial Block Face Scanning Electron Microscopy of a human glomerulus. A podocyte cell body on capillaries after segmentation in 3D projection. Purple/Pink = Capillaries. Green = Podocyte cell body. Yellow is SPS under the main cell body. Scale bar = 10μm. Click here for file

Additional file 8 **Video S4D.** Serial Block Face Scanning Electron Microscopy of a human glomerulus. As **Video S4C** without the capillaries. Green = Podocyte cell body. Yellow is SPS under the main cell body. Scale bar = 10μm. Click here for file

Additional file 9 **Video S5A.** Micrographs of the Human Glomerulus demonstrating interpodocyte space. 300 Slice (150nm) sequence. Scale Bar = 10μm. Click here for file

Additional file 10 **Video S5B.** Micrographs of the Human Glomerulus demonstrate interpodocyte space. As **Video S5B** but with interpodocyte space segmented (yellow) and highlighted. Scale Bar = 10μm. Click here for file

Additional file 11 **Video S5C.** Micrographs of the Human Glomerulus demonstrate interpodocyte space. The extracted IPS as a 3D volume. The brown arrows point to the 2 connections with larger IPS. One is a substantial connection (circa 5μm wide, though filled with podocyte cell bodies), but the other connection is highly tortuous and very narrow if it connects at all. Between the two ‘bulbs’ of IPS is a narrow (<1.5μm) neck (blue arrow) and it is tortuous with parts of podocytes intervening. Scale Bar = 10μm. Click here for file

Additional file 12 **Video S6A.** FIB-SEM of a glomerular capillary filtration barrier. 280 Slice (10nm) sequence of realigned micrographs. Scale Bars= 1μm. Click here for file

Additional file 13 **Video S6B.** FIB-SEM of a glomerular capillary filtration barrier. 3D volume view of the barrier and podocyte. Scale Bars= 1μm. Click here for file

Additional file 14 **Video S7A.** Transmission electron tomography of 300nm thick section of glomerular capillary glycocalyx. The tilt angle (single tilt shown) micrographs aligned for reconstruction. Gold alignment markers are visible on the surface. Scale Bar=150nm. Click here for file

Additional file 15 **Video S7B.** Transmission electron tomography of 300nm thick section of glomerular capillary glycocalyx. z sequence of the reconstruction. Scale Bar=150nm. Click here for file

Additional file 16 **Video S7C.** Transmission electron tomography of 300nm thick section of glomerular capillary glycocalyx. 3D volume view of the reconstruction. Scale Bar=150nm. Click here for file

## References

[B1] Deen WM Lazzara MJ Myers BD Structural determinants of glomerular permeability Am J Physiol Renal Physiol 2001 281 F579 F596 11553505 10.1152/ajprenal.2001.281.4.F579

[B2] Salmon AHJ Neal CR Harper SJ New aspects of glomerular filtration barrier structure and function: five layers (at least) not three Curr Opin Nephrol Hypertens 2009 18 197 205 19365184 10.1097/MNH.0b013e328329f837

[B3] Avasthi PS Koshy V Glomerular endothelial glycocalyx Contrib Nephrol 1988 68 104 113 3233988 10.1159/000416500

[B4] Avasthi PS Koshy V The anionic Matrix at the rat glomerular enodothelial surface Anat Rec 1988 220 258 266 10.1002/ar.1092200306 2966599

[B5] Rostgaard J Qvortrup K Sieve plugs in fenestrae of glomerular capillaries - site of the filtration barrier? Cells Tissues Organs 2002 170 132 138 10.1159/000046186 11731701

[B6] Haraldsson B Nystrom J Deen WM Properties of the glomerular barrier and mechanisms of proteinuria Physiol Rev 2008 88 451 487 10.1152/physrev.00055.2006 18391170

[B7] Jeansson M Haraldsson B Glomerular size and charge selectivity in the mouse after exposure to glucosaminoglycan-degrading enzymes J Am Soc Nephrol 2003 14 1756 1765 10.1097/01.ASN.0000072742.02714.6E 12819235

[B8] Jeansson M Haraldsson B Morphological and functional evidence for an important role of the endothelial cell glycocalyx in the glomerular barrier Am J Physiol Renal Physiol 2006 290 F111 F116 16091582 10.1152/ajprenal.00173.2005

[B9] Salmon AHJ Ferguson JK Burford JL Gevorgyan H Nakano D Harper SJ Bates DO Peti-Peterdi J Loss of the Endothelial Glycocalyx Links Albuminuria and Vascular Dysfunction J Am Soc Nephrol 2012 23 8 1339 1350 10.1681/ASN.2012010017 22797190 PMC3402289

[B10] Neal CR Crook H Bell E Harper SJ Bates DO Three-dimensional reconstruction of glomeruli by electron microscopy reveals a distinct restrictive urinary subpodocyte space J Am Soc Nephrol 2005 16 1223 1235 10.1681/ASN.2004100822 15829713

[B11] Salmon AHJ Toma I Sipos A Muston PR Harper SJ Bates DO Neal CR Peti-Peterdi J Evidence for restriction of fluid and solute movement across the glomerular capillary wall by the subpodocyte space Am J Physiol Renal Physiol 2007 293 F1777 F1786 10.1152/ajprenal.00187.2007 17804486

[B12] Neal CR Muston PR Njegovan D Verrill R Harper SJ Deen WM Bates DO Glomerular filtration into the subpodocyte space is highly restricted under physiological perfusion conditions Am J Physiol Renal Physiol 2007 293 F1787 F1798 10.1152/ajprenal.00157.2007 17715264

[B13] Gautier A Bernhard W Oberling C *sur lexistence dun appareil lacunaire peri-capillaire du glomerule de malpighi, revelee par le microscope electronique Comptes Rendus Des Seances De La Societe De Biologie Et De Ses Filiales 1950 144 1605 1607 14822344

[B14] Elias H Allara E Elias PM Murthy AS The podocytes, re-examined Zeitschrift fur mikroskopisch-anatomische Forschung 1965 72 344 365 5826826

[B15] Neal CR Harper SJ Bates D Abstract: 3-D reconstruction of the glomerular barrier and overlying podocyte reveals a sub-podocyte space: a new urinary space within the glomerulus? J Physiol 2003 552P C26

[B16] Oltean S Neal CR Sison K Qui Y Patel P Ahad T Alsop C Lee T Harper SJ Bates DO VEGF165b overexpression restores normal glomerular filtration barrier function in VEGF164-overexpressing adult mice Am J Physiol in review 2012 303 7 F1026 F1036 10.1152/ajprenal.00410.2011 PMC3469682 22811490

[B17] Arkill KP Neal CR Mantell JM Michel CC Qvortrup K Rostgaard J Bates DO Knupp C Squire JM 3D reconstruction of the glycocalyx structure in mammalian capillaries using electron tomography Microcirculation 2012 19 343 351 10.1111/j.1549-8719.2012.00168.x 22324320 PMC3808814

[B18] West JB Fu ZX Deerinck TJ Mackey MR Obayashi JT Ellisman MH Structure-function studies of blood and air capillaries in chicken lung using 3D electron microscopy Respir Physiol Neurobiol 2010 170 202 209 10.1016/j.resp.2009.12.010 20038456 PMC2821748

[B19] Denk W Horstmann H Serial block-face scanning electron microscopy to reconstruct three-dimensional tissue nanostructure PLoS Biol 2004 2 1900 1909 10.1371/journal.pbio.0020329 PMC524270 15514700

[B20] Mantell JM Verkade P Arkill KP Iop Quantitative Biological Measurement In Transmission Electron Tomography Electron Microscopy And Analysis Group Conference 2012 371 Journal of Physics Conference Series

[B21] Luther PK Lawrence MC Crowther RA A method for monitoring the collapse of plastic sections as a function of electron dose Ultramicroscopy 1988 24 7 18 10.1016/0304-3991(88)90322-1 3344552

[B22] Kremer JR Mastronarde DN McIntosh JR Computer visualization of three-dimensional image data using IMOD J Struct Biol 1996 116 71 76 10.1006/jsbi.1996.0013 8742726

[B23] Mastronarde DN Dual-axis tomography: an approach with alignment methods that preserve resolution J Struct Biol 1997 120 343 352 10.1006/jsbi.1997.3919 9441937

[B24] Schneider CA Rasband WS Eliceiri KW NIH Image to ImageJ: 25 years of image analysis Nature Methods 2012 9 671 675 10.1038/nmeth.2089 22930834 PMC5554542

[B25] Schindelin J Arganda-Carreras I Frise E Kaynig V Longair M Pietzsch T Preibisch S Rueden C Saalfeld S Schmid B Fiji: an open-source platform for biological-image analysis Nat Methods 2012 9 676 682 10.1038/nmeth.2019 22743772 PMC3855844

[B26] Arkill KP Knupp C Michel CC Neal CR Qvortrup K Rostgaard J Squire JM Similar endothelial glycocalyx structures in microvessels from a range of mammalian tissues: evidence for a common filtering mechanism? Biophys J 2011 101 1046 1056 10.1016/j.bpj.2011.07.036 21889441 PMC3164174

[B27] Squire JM Chew M Nneji G Neal C Barry J Michel C Quasi-periodic substructure in the microvessel endothelial glycocalyx: a possible explanation for molecular filtering? J Struct Biol 2001 136 239 255 10.1006/jsbi.2002.4441 12051903

[B28] Hawes P Netherton CL Mueller M Wileman T Monaghan P Rapid freeze-substitution preserves membranes in high-pressure frozen tissue culture cells J Microsc-Oxford 2007 226 182 189 10.1111/j.1365-2818.2007.01767.x 17444947

[B29] Bushby A Mariggi G Armer H Collinson L Correlative light and volume electron microscopy: using focused ion beam scanning electron microscopy to image transient events in model organisms Meth Cell Biol 2012 111 357 482 10.1016/B978-0-12-416026-2.00018-2 22857937

