# Supplementary material for: Resolution of the three dimensional structure of components of the glomerular filtration barrier
Source: BMC Nephrol. 2014 Feb 1;15:24. doi: 10.1186/1471-2369-15-24 (PMC3922634; doi:10.1186/1471-2369-15-24)
Supplement: Additional file 1 — Supplementary Material. Additional heavy metal staining of human blocks. [file 1471-2369-15-24-S1.docx]

**Additional file 16**

**Supplementary Material Staining of Human Blocks**

The vasculature of an unused transplant kidney was pre-perfused with Marshall’s transport solution[[1](#_ENREF_1), [2](#_ENREF_2)] and the kidney transported on ice. Renal arteries were cannulated and perfused (100mmHg hydrostatic pressure) with a Mammalian HEPES Ringer containing Ficoll 400 as an oncotic balance (25mmHg Colloid Osmotic pressure). This was replaced with a 2.5% glutaraldehyde Mammalian HEPES Ringer solution at the same (human) hydrostatic and oncotic pressures.

SBF-SEM specific preparation: For increased contrast extra heavy metal staining steps are required compared to standard transmission electron microscopy. This is partly due to SBF-SEM imaging the block surface which is unstained by EM section stains used to enhance contrast, but primarily due to the current inability of the Gatan system to use improved backscatter detectors. This technique has been used by other authors [[3-5](#_ENREF_3)]but modified by us. 1mm diameter pieces were treated with a metal fixation and staining regime as below:

1. 2mM CaCl_2_ in 0.1M Sodium Cacodylate buffer (5 x 5 min, 4ºC).
2. 1% Osmium tetroxide (OsO_4_) in 0.1M Cacodylate buffer (8 hours, 4ºC)
3. 0.1M Cacodylate washes. (3 x 10 min, RT)
4. 1.5% Potassium ferrocyanide, 1% OsO_4_, 2mM CaCl_2_ in 0.1M Cacodylate buffer (1 hr, 4ºC)
5. Dist. water washes ( 4 x 10mins, RT)
6. 1% thiocarbohydrazide in dist. Water (30 min, RT)
7. Dist. water washes ( 4 x 10mins, RT)
8. 2% OsO_4_ in dist. Water (1hr, 4ºC)
9. Dist. water washes ( 4 x 10mins, RT)
10. 2% Uranyl acetate in dist. Water (10hrs, 4ºC, dark)
11. Dist. water washes ( 4 x 10mins, RT)
12. Walton’s Lead aspartate (30mins, RT)
13. Dist. water washes ( 4 x 10mins, RT)
14. Dehydrate graded ethanol
15. 100% ethanol (3 x 15 mins, RT)
16. Propylene oxide (2 x 10 mins, RT)
17. Propylene oxide: Araldite resin mix, 1:1 (2-3 hrs, RT)
18. Araldite resin mix (2 x ≥3 hrs)
19. Embed and polymerise (60ºC 24hrs)

The Araldite resin mix was made to a hard formulation better suited to the cutting and imaging characteristics of an SBF-SEM machine. Toluidine Blue stained 0.5µm resin sections were used to find glomeruli in the embedded block.

1. Nicholson ML, Metcalfe MS, White SA, Waller JR, Doughman TM, Horsburgh T, Feehally J, Carr SJ, Veitch PS: **A comparison of the results of renal transplantation from non-heart-beating, conventional cadaveric, and living donors.** *Kidney International* 2000, **58:**2585-2591.

2. Marshall VC: **Renal preservation.** In *Kidney Transplantation: Principles and Practice.* 5th edition. Edited by Morris PJ. Philadelphia: Saunders; 2001: 113-134

3. Denk W, Horstmann H: **Serial block-face scanning electron microscopy to reconstruct three-dimensional tissue nanostructure.** *Plos Biology* 2004, **2:**1900-1909.

4. West JB, Fu ZX, Deerinck TJ, Mackey MR, Obayashi JT, Ellisman MH: **Structure-function studies of blood and air capillaries in chicken lung using 3D electron microscopy.** *Respiratory Physiology & Neurobiology* 2010, **170:**202-209.

5. Williams ME, Wilke SA, Daggett A, Davis E, Otto S, Ravi D, Ripley B, Bushong EA, Ellisman MH, Klein G, Ghosh A: **Cadherin-9 Regulates Synapse-Specific Differentiation in the Developing Hippocampus.** *Neuron* 2011, **71:**640-655.
